# Supplementary figures and images for: Is intergenerational elasticity (IGE) a misleading measure of wealth mobility?
Source: PLoS One. 2025 May 29;20(5):e0324266. doi: 10.1371/journal.pone.0324266 (PMC12121768; doi:10.1371/journal.pone.0324266)

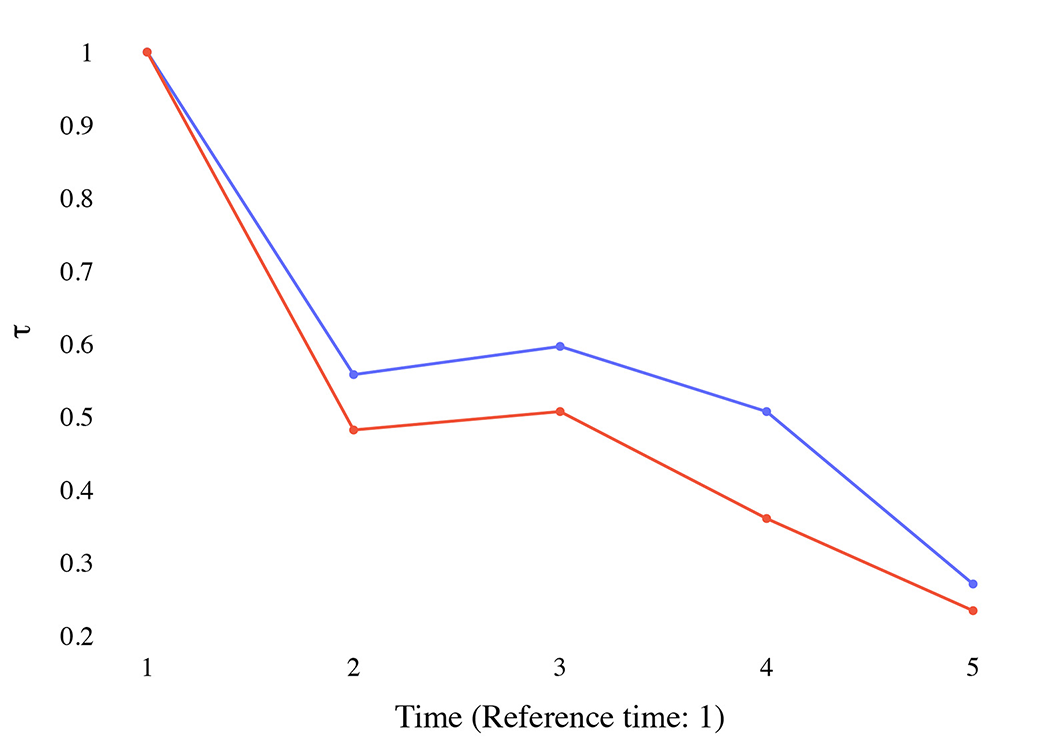

Supplement: S1 Fig — If one aims to observe the evolution of τ over periods, the researcher may consider a subgroup to aggregate the individual wealth. With the individual wealth dataset from [11], the wealth values are aggregated for surnames. Here, to be able to have the same length of the data at each period, we only consider the surnames that have data points for all five periods, resulting in 88 surnames over the five periods, i.e., 440 observations. Then, Kendall’s tau is calculated for each period, with the first period as the reference time to compare. The log wealth values of individuals of a surname are aggregated in two ways: median and mode. The blue line connects the τ values calculated with the surname’s mean log wealth values, and the red line connects the τ values calculated with the surname’s median log wealth values. τ=1 at the first period since the first period’s log wealth rank is the reference, and the value quickly decreases over time. (TIFF) [file pone.0324266.s001.tif]
