# Supplementary material for: Is intergenerational elasticity (IGE) a misleading measure of wealth mobility?
Source: PLoS One. 2025 May 29;20(5):e0324266. doi: 10.1371/journal.pone.0324266 (PMC12121768; doi:10.1371/journal.pone.0324266)
Supplement: S1 Appendix — In the following, we show the wealth dynamics implied in the IGE model by expressing the model with respect to the initial wealth, the convergence value, and the slope. (TEX, PDF) [file pone.0324266.s002.pdf]

Is Intergenerational Elasticity (IGE) a Misleading Measure of Wealth Mobility?

Seorin Kim[1,2*], Arne Vanhoyweghen[1], Wouter Ryckbosch[2], Vincent Ginis[1]

1 Data Analytics Laboratory, Vrije Universiteit Brussel, Brussels, Belgium
2 Social History of Capitalism, Vrije Universiteit Brussel, Brussels, Belgium

* Corresponding author
Email: seorin.kim@vub.be (SK)

A Wealth dynamics implied in the IGE model

In the following, we show the wealth dynamics implied in the IGE model by expressing the model with respect to the initial wealth, the convergence value, and the slope.

An AR(1) model assumes that the expectation of the true population's wealth is the same for all generations (i.e., $E[\ln W_t] = E[\ln W_{t-1}] = \mu, \forall t \geq 1$) [1]. Notice that since the logarithm of wealth is used, the wealth is restricted to positive values, leading e^{μ} to be positive while μ can be negative. Usually, a first-order autoregressive process is stable with the mean μ when $|\beta| < 1$ [1,2]. Assuming the equal expected log wealth over time or simply called population mean, μ, the intercept, $\alpha = \mu(1-\beta)$ from the following:

$$\begin{aligned} \ln W_{it} - \mu &= \beta(\ln W_{it-1} - \mu) + \varepsilon_{it} \\ \ln W_{it} &= \mu(1-\beta) + \beta \ln W_{it-1} + \varepsilon_{it} \end{aligned}$$

With $\alpha = \mu(1-\beta)$,

$$\ln W_{it} = \alpha + \beta \ln W_{it-1} + \varepsilon_{it}$$

Utilizing the autocorrelation properties on the population regression line of the model,

$$\begin{aligned} \ln W_{it} &= \alpha + \beta \ln W_{it-1} \\ &= \alpha + \beta(\alpha + \beta \ln W_{it-2}) \\ &= \alpha + \beta\alpha + \beta^2 \ln W_{it-2} \\ &\vdots \\ &= \beta^t \ln W_{i0} + \beta^{t-1}\alpha + \beta^{t-2}\alpha + \cdots + \beta\alpha + \alpha \end{aligned}$$

When $|\beta| < 1$, the series converges to the following,

$$\ln W_{it} = \beta^t \ln W_{i0} + (1-\beta^t)\mu$$

This known conversion of the geometric series comes from the following,

$$\begin{aligned} S &= \beta^{t-1}\alpha + \beta^{t-2}\alpha + \cdots + \beta\alpha + \alpha \\ \beta S &= \beta^t\alpha + \beta^{t-1}\alpha + \cdots + \beta^2\alpha + \beta\alpha \\ (1-\beta)S &= (1-\beta^t)\alpha \\ S &= (1-\beta^t)\mu \end{aligned}$$

To interpret in terms of wealth, not the logarithm of wealth, the equation can be exponentiated,

$$W_{it} = e^{\alpha\frac{(1-\beta^t)}{1-\beta}} W_{i0}^{\beta^t} = e^{(1-\beta^t)\mu} W_{i0}^{\beta^t}$$

which suggests positive β given the exponential term in $W_{i0}^{\beta^t}$ and $|\beta| < 1$ considering the geometric series rule and the equation expressed in terms of α. Here, it is observable that wealth depends on both α and β.

Examining the wealth dynamics in the limit, when $0 < \beta < 1$,

$$\lim_{t\to+\infty} W_{it} = e^{\mu}$$

And when $\beta = 0$,

$$W_{it} = e^{\mu}$$

And when $\beta > 1$, W_{it} does not converge to μ as we are working with the logarithm of wealth.

References

1. Shumway RH, Stoffer DS. Time Series Analysis and Its Applications: With R Examples. Springer Texts in Statistics. Springer International Publishing; 2017. Available from: `https://books.google.be/books?id=sfFdDwAAQBAJ`.

2. Le Breton A, Pham DT. On the bias of the least squares estimator for the first order autoregressive process. Annals of the institute of Statistical Mathematics. 1989;41:555–563.
